# Therapeutic use of a receptor mimic probiotic reduces intestinal Shiga toxin levels in a piglet model of hemolytic uremic syndrome

**DOI:** 10.1186/1756-0500-7-331

**Published:** 2014-06-02

**Authors:** Shannon J Hostetter, Amy F Helgerson, James C Paton, Adrienne W Paton, Nancy A Cornick

**Affiliations:** 1 Department of Veterinary Pathology, Iowa State University, 1600 S. 16th Street, Ames, IA 50011-1250, USA; 2 Department of Veterinary Microbiology and Preventive Medicine, Iowa State University, 1600 S. 16th Street, Ames, IA 50011-1250, USA; 3 Research Centre for Infectious Diseases, School of Molecular and Biomedical Science, University of Adelaide, Adelaide 5005, South Australia; 4 Current address – Department of Environmental Health and Safety, Iowa State University, Ames, IA 50011, USA

**Keywords:** Shiga toxin-producing *Escherichia coli*, Probiotic, Therapy, Hemolytic uremic syndrome, Edema disease, Animal model

## Abstract

**Background:**

Hemolytic uremic syndrome (HUS) is a systemic and potentially fatal complication of gastroenteritis secondary to Shiga toxin-producing enterohemorrhagic *Escherichia coli* (EHEC) infection characterized by microangiopathic hemolytic anemia, thrombocytopenia, and acute renal damage. Shiga toxin (Stx), the toxin principle in HUS, is produced locally within the gut following EHEC colonization and is disseminated via the vasculature. Clinical development of HUS currently has no effective treatment and is a leading cause of renal failure in children. Novel post-exposure therapies are currently needed for HUS; therefore, the purpose of this study was to investigate the efficacy of a Stx receptor mimic probiotic in a porcine model of HUS. Edema disease, an infection of swine caused by host adapted Shiga toxin-producing *Escherichia coli* (STEC) and mediated by Shiga toxin 2e (Stx2e), shares many pathogenic similarities to HUS. In this study, three-week old piglets were inoculated with STEC and 24 hours later treated twice daily with a probiotic expressing an oligosaccharide receptor mimic for Stx2e to determine if the probiotic could reduce intestinal toxin levels.

**Methods:**

Piglets were orally inoculated with 10^10^ CFU of STEC strain S1191 eight days after weaning. Beginning day 1 post-inoculation, piglets were treated orally twice daily with 5 × 10^11^ CFU of either the receptor mimic probiotic or a sham probiotic for 10 days. Intestinal Stx2e levels were assessed daily via Vero cell assay. The efficacy of the probiotic at reducing intestinal Stx2e, vascular lesions, and clinical disease was evaluated with repeated measures ANOVA and Fisher’s exact test as appropriate.

**Results:**

The probiotic significantly reduced intestinal Stx2e, as reflected by decreased fecal toxin titers on days 3–8 post-inoculation (p < 0.01). Despite this reduction in intestinal toxin levels, however, the probiotic failed to reduce the incidence of vascular necrosis in target organs and had no effect on clinical disease.

**Conclusions:**

The data suggest that post-exposure treatment with a Stx-binding probiotic is effective in reducing intestinal toxin burden. Future studies could target this approach for possible development of post-exposure interventions.

## Background

Enterohemorrhagic *Escherichia coli* (EHEC), classically of serotype O157:H7, cause sporadic cases as well as outbreaks of food borne illness characterized by hemorrhagic colitis [1]. Ruminants are the primary reservoir for EHEC, and contamination of either produce or meat with fecal matter from carriers serve as the primary source of infection [2]. A small percentage of patients (typically 5-15%) with diarrhea secondary to EHEC infection develop a systemic and potentially fatal complication known as hemolytic uremic syndrome (HUS), characterized by the triad of microangiopathic hemolytic anemia, thrombocytopenia, and acute renal damage. Historically, HUS has been more prevalent in children under the age of 10 and the elderly, and it remains the leading cause of renal failure in children in the United States [1,3-5]. More recently, a large outbreak of gastroenteritis and HUS in Germany caused by an atypical enteroaggregative *E. coli* that produced Shiga toxin (serotype O104:H4), was characterized by a higher prevalence of HUS in adults versus children. In this particular outbreak, 88% of the patients who developed HUS were adults [6-8]. At present, there is no known method to predict which patients with diarrhea caused by EHEC will develop HUS, and no single effective treatment for the disorder once it is diagnosed. Currently, HUS treatment involves fluid therapy, dialysis, and management of coagulation disorders; antimicrobial therapy, however, remains controversial [9-11].

Although the pathogenesis is multifactorial, Shiga toxin (Stx) produced by EHEC is the principal mediator of HUS. EHEC that produce Stx1, Stx2, or both toxins have been associated with HUS development. Epidemiologic evidence and data from animal models show that strains producing Stx2 only are more likely to cause severe disease [12,13]. The preferred receptor for Stx1, Stx2, and most of its variants is globotriaosylceramide (Gb3); globotetraosylceramide (Gb4) is the preferred receptor of Stx2e, the Stx2 variant which is primarily associated with edema disease of swine [14]. Following EHEC colonization, Stx is produced locally within the intestine, and then crosses the intestinal mucosa and vascular endothelium to gain access to the bloodstream. Although the exact mechanism for systemic absorption of Stx is unknown, recent evidence suggests absorption across the epithelium occurs via a transcellular pathway and is increased under microaerobic conditions [15]. Once the toxin reaches the blood, it binds the receptor on the surface of vascular endothelial cells in key target organs (kidney, brain) and is internalized [16]. Toxin internalization within endothelial cells results in inhibition of protein synthesis, leading to cell death [4].

Many experimental approaches to the treatment of EHEC infection and/or HUS have been explored. One potential therapeutic approach for HUS is to neutralize the activity of Stx and several compounds have been developed for that purpose, including oral synthetic receptor mimics and specific anti-Stx antibody [17-20]. In this fashion, a probiotic that expresses Stx receptor mimics on its surface was developed for treatment of active EHEC infection [21]. The advantage of this particular probiotic is its ability to bind and absorb Stx within the intestinal lumen, therefore potentially increasing its utility as a post-exposure therapeutic agent. The probiotic is a recombinant *E. coli* R1 strain (CWG308) that contains a plasmid (pJCP-Gb3) encoding two *Neisseria sp.* galactosyltransferase genes that when expressed, create a cell surface mimic of the Stx receptor. The binding capacity of this recombinant strain for either Stx1 or Stx2 is approximately 10,000× greater than that of SYNSORB Pk (a synthetic carbohydrate receptor mimic previously developed for treatment of STEC infection), and this efficacy has been demonstrated by several *in vivo* protection studies in mice [19,21-23]. This particular construct was effective at neutralizing most of the Stx2 variants; however, it was less efficacious against Stx2e, which binds preferentially to Gb4. As a result, a new recombinant strain that expressed surface Gb4 was created as a potential probiotic strain for use in the prevention of edema disease of swine [22].

Edema disease, an infection of weaned swine caused by host-adapted strains of *E. coli*, has been adapted by our laboratory as an animal model of systemic EHEC disease in human beings [24,25]. The edema disease model has several advantages over other models of HUS, the most outstanding of which is that it is a naturally occurring disease. Edema disease and HUS share similar early events in their pathogeneses, including localized production of toxin in the gut, toxin translocation across gut epithelium, and dissemination to target organs via the bloodstream [24,25]. Additionally, as in human EHEC infections, not all Shiga-toxin producing *Escherichia coli* (STEC) inoculated swine develop systemic disease; the majority of pigs undergo an uncomplicated recovery from the diarrhea phase. Other advantages include a similar time course of pathogenic events manifested by a prodromal diarrhea phase with delayed development of systemic complications [24]. In this model, approximately 30% of STEC challenged swine succumb to clinical disease secondary to Shiga toxemia, characterized by subcutaneous edema, neurological signs and/or sudden death.

We hypothesized that treatment with the probiotic expressing surface globotetraose would bind and trap Stx2e within the intestinal lumen, thereby reducing systemic toxin absorption and preventing clinical edema disease. The primary purpose of this study was to assess the ability of this Stx receptor–mimic probiotic to reduce fecal Shiga toxin in a naturally occurring STEC infection when administered during active infection. A secondary goal was to test the efficacy of the probiotic at preventing clinical disease.

## Methods

### Bacterial strains

S1191 is an STEC strain isolated from a pig with edema disease. This strain belongs to serotype O139, is resistant to chloramphenicol, and produces Stx2e, hemolysin, F18ab fimbriae and heat-stable enterotoxin B. Strain 123 is a non-pathogenic *E. coli*, serogroup O143, isolated from a healthy pig. Inocula were prepared as described previously [24].

Creation of the probiotic strain, an *E. coli* R1 derivative expressing surface Gal*N*Acβ(1 → 3)Galα(1 → 4)Galβ(1 → 4)Glc (globotetraose), has been described in detail previously [22]. Briefly, three *Neisseria sp.* glycosyltransferase genes (*lgtC, lgtE,* and *lgtD*) and a UDP-Gal*N*Ac-4-epimerase gene (*gne*) were cloned into the plasmid vector pK184 to create plasmid pGb4 and inserted into a derivative of *E. coli* R1 (CWG308). This strain has a *waaO* mutation in the outer core lipopolysaccharide (LPS) biosynthesis locus that results in production of a truncated LPS core terminating in glucose. Expression of the plasmid-encoded glycosyltransferase genes results in linkage of Gal*N*Acβ(1 → 3)Galα(1 → 4)Galβ(1 → 4) onto the terminal glucose. The sham strain is the *E. coli* derivative R1 (CWG308) with the plasmid vector pK184 only. The treatment and sham strains were grown overnight in tryptic soy broth (TSB) plus kanamycin (30 μg/ml) and IPTG (20 μg/ml), concentrated 100× and re-suspended in TSB containing 10% NaCO_3,_ 20% sucrose and 21% glycerol. Treatments were stored at -80C and thawed just prior to use.

### Reproduction of clinical edema disease

Animal experiments were carried out in accordance with the Iowa State University Animal Care and Use Committee. The study design proposed here to reproduce clinical edema disease follows a previously described protocol and is outlined in Table 1[24]. Briefly, two week-old crossbred pigs were acclimated to a high soy protein diet for one week prior to challenge [26]. Pigs were orally inoculated with 10^10^ CFU of STEC strain S1191 eight days after weaning. Control pigs received 10^10^ CFU of the non-pathogenic *E. coli* strain 123. Separate rooms in the same housing facility were used for each treatment group. Pigs were monitored twice daily for clinical signs of edema disease (subcutaneous edema, recumbency or neurological disturbances such as ataxia, circling or sudden death). Pigs that developed neurological signs were euthanized by an intravenous overdose with barbiturate. Pigs that failed to develop neurological signs were euthanized at the termination of the study (14 days post-inoculation). All pigs underwent a complete necropsy, with inspection of major organs for gross lesions and collection of tissue samples stored in 10% neutral buffered formalin taken from ileum (section taken approximately 1 m proximal to ileocecal valve) and brainstem (section taken at rostral medulla) for histology. Fixed tissues were embedded in paraffin and sectioned at 5 μm. Histologic evaluation of tissues for vascular lesions consistent with edema disease followed an established protocol [24,27]. Briefly, hematoxylin and eosin stained sections of ileum and brainstem were examined by a board certified veterinary pathologist (SJH) blinded to the treatment groups for qualitative evidence of vascular necrosis in arterioles. Scores were considered positive if two or more necrotic vessels were identified per complete tissue section.

**Table 1:** Experimental design to assess the effect of a probiotic expressing a Shiga toxin receptor mimic on the incidence of edema disease

**Experimental group**	**Number of pigs**^**a**^	**Pig age at inoculation**	**Inoculation strain**	**Treatment**
Probiotic	30	24-26 days	S1191^c^	CWF308 (pJCPGb_4_)
Sham	30	24-26 days	S1191^c^	CWF308
Negative control	10	24-26 days	123^b^	None

^a^Each group represents the cumulative number of pigs from three separate experimental replications. ^b^Non-pathogenic *E. coli* strain. ^c^STEC strain.

Three week old piglets were orally inoculated with either pathogenic STEC strain 1191 (probiotic and sham groups) or non-pathogenic *E. coli* strain 123 (negative control group). STEC inoculated pigs were treated with either a Shiga toxin 2e receptor mimic probiotic (probiotic group) or a sham probiotic strain which lacked the receptor mimic (sham group). Treatment was administered twice daily, and was initiated 24 hours post-inoculation with the STEC strain.

### Treatment with the probiotic

Pigs inoculated with STEC strain S1191 were treated with either *E. coli* CWG308 containing the plasmid pGb4 (probiotic) or CWG308 containing the vector pK184 only (sham). Individual pigs were orally dosed with 5 × 10^11^ CFU of either strain twice daily for 10 days beginning day 1 post-inoculation.

### Determination of Stx2e in feces

Feces were collected daily from control and STEC-inoculated pigs on days 1–10 post-inoculation, and stored at 4°C until processing. Stx2e concentrations were assessed using a Vero cell assay as described previously [24]. Briefly, samples were diluted two-fold in PBS and added to monolayers of Vero cells in microtiter plates. Toxin titers were expressed as the log of the reciprocal of the greatest dilution that resulted in ≥ 50% Vero cell death. Specificity of the titers for Stx2e was confirmed by neutralization with anti-Stx2e antibody.

### Statistical analysis

Comparison of fecal toxin titers was performed using a two way repeated measures ANOVA with an unweighted means analysis (Prism, Graphpad software, Inc, La Jolla, CA). Bonferroni post hoc test was used to compare group differences on specific days. For all analyses, a P value < 0.05 was considered statistically significant. Incidences of vascular lesions and clinical disease between probiotic and sham groups were compared using Fisher’s exact test.

## Results

### Bacterial colonization

STEC strain 1191 was recovered from feces of all challenged pigs (probiotic and sham groups) at both 2 and 5 days post inoculation. None of the negative control pigs were positive for STEC at either time-point. The probiotic *E. coli* strain CWG308 (pJCPGb_4_) was recovered from all pigs in the probiotic treated group at both 2 and 5 days post inoculation.

### Fecal toxin titers

**Figure 1:**
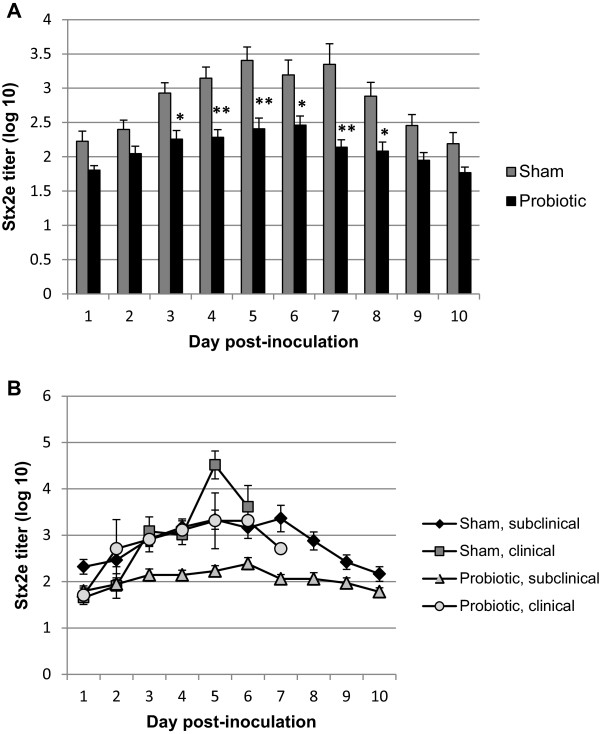
**Effect of twice daily treatment with a probiotic expressing globotetraose, initiated 24 hours post-inoculation with Shiga toxin-producing *Escherichia coli*, on mean fecal Shiga toxin 2e titers in piglets.** Globotetraose is the preferred receptor for Stx2e. **A)**. Treatment with the probiotic significantly reduced titers compared to sham treated pigs on days 3–8 post inoculation (*, P < 0.01, **P < 0.001, repeated measures ANOVA with Bonferroni post hoc test). Error bars indicate SEM. **B)**. Although fecal Shiga toxin levels were reduced overall within the probiotic group, pigs that developed clinical edema disease had higher levels of fecal Shiga toxin, similar to the sham treated group.

Stx titers were measured daily in feces to assess the efficacy of the probiotic at adsorbing Stx within the intestinal lumen. Increases in fecal Stx titers in both the sham and probiotic treated STEC inoculated groups were detectable beginning day 1 post inoculation, and peaked on days 5–7 post inoculation (Figure 1A). Treatment with the probiotic significantly reduced fecal Stx2e levels compared to the sham treated group on days 3–8 post-inoculation (p < 0.01, repeated measures ANOVA with Bonferroni post hoc test, Figure 1A). None of the control pigs had detectable Stx2e in their feces at any time point throughout the study (data not shown).

### Clinical disease

Seven out of sixty pigs inoculated with the STEC strain 1191 developed clinical edema disease and no differences were clinically detected between probiotic or sham treated pigs (Table 2). Of the pigs with clinical edema disease, three exhibited neurological signs prior to euthanasia and four were found dead. In summary, three of the seven clinically affected pigs were in the probiotic treatment group, and four were in the sham group resulting in an overall disease incidence of 10% in the probiotic group and 13% in the sham group. None of the ten pigs inoculated with the non-pathogenic *E. coli* strain 123 developed clinical signs of edema disease or died.

**Table 2:** **Incidences of clinical disease and vascular lesions in pigs inoculated with Shiga toxin-producing *Escherichia coli *** **and treated with a probiotic strain expressing a Shiga toxin receptor mimic vs. a sham strain lacking the receptor mimic**

**Experimental group**	**Number of pigs**^**d**^	**Incidence of clinical disease**	**Incidence of vascular lesions in brainstem**	**Incidence of vascular lesions in ileum**
Probiotic^a^	30	3/30	11/30	17/30
Sham^b^	30	4/30	17/30	19/30
Negative control^c^	10	0/10	0/10	0/10

^a^Inoculated with STEC strain, treated with probiotic. ^b^Inoculated with STEC strain, treated with sham. ^c^Inoculated with non-pathogenic *E. coli* strain, no treatment. ^d^Sum of three experimental replicates.

Negative control pigs were inoculated with a non-pathogenic, commensal *E.coli* strain. Probiotic treatment had a tendency to reduce the incidence of vascular lesions in brainstem (p = 0.098); however, treatment with the receptor mimic probiotic did not significantly reduce the incidences of clinical disease or vascular lesions (Fisher’s exact test).

### Comparison of fecal toxin titers between clinical and non-clinical pigs

Fecal toxin titers in clinical pigs were compared with titers in non-clinical pigs in both the sham and probiotic treatment groups (Figure 1B). Since all pigs that developed edema disease fell ill prior to day 8 post inoculation, fecal titers are not available for these pigs beyond day 7 post inoculation. Although treatment with the probiotic reduced fecal toxin titers overall, those pigs treated with the probiotic that developed clinical disease had, on average, toxin titers similar to the sham treated group.

### Vascular lesions

**Figure 2:**
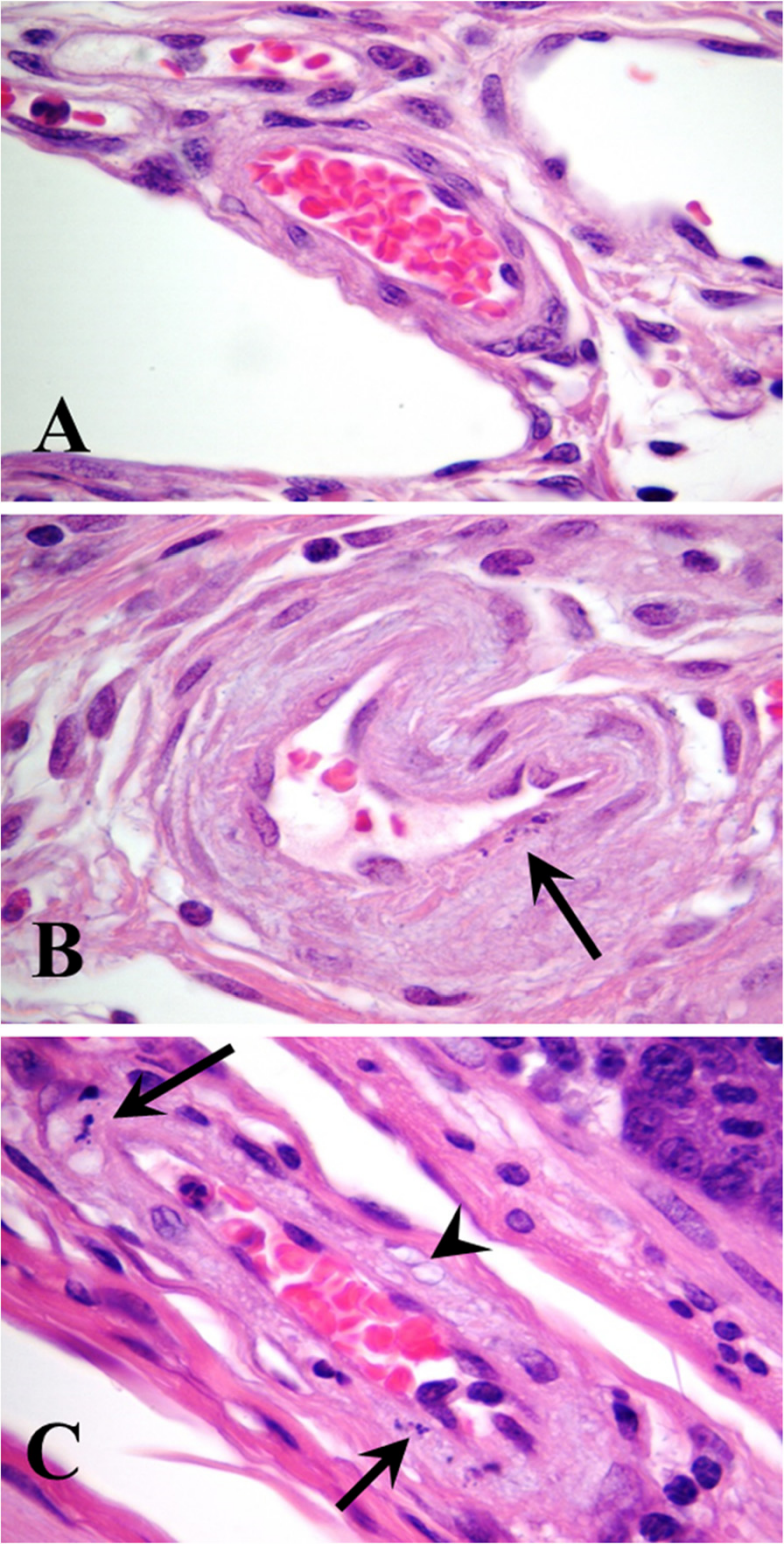
**Histological confirmation of vascular injury secondary to Shiga toxemia in ilea (submucosa).** Arterioles in control pigs inoculated with non-pathogenic *Escherichia coli* lacked lesions **(A)**. Arteriole from pig inoculated with Shiga toxin producing *E. coli* (STEC) strain S1191 and treated with the sham probiotic strain, which lacked the receptor Shiga toxin mimic **(B)**. Segmental changes within the tunica media characterized by the presence of karyorrhectic nuclear debris (arrow) from a myocyte. Arteriole from pig inoculated with STEC strain S1191 and treated with the receptor mimic probiotic **(C)**. Note nuclear remnants (arrows) of myocytes within the tunica media, as well as vacuolization (arrowhead) of the sarcoplasm. Hematoxylin and eosin, ×1000 magnification.

Brainstem and ileum were assessed postmortem for vascular evidence of Shiga toxemia. Histologic evidence of edema disease (vascular necrosis in two or more vessels per tissue section) was not detected in any of the ten pigs inoculated with the non-pathogenic *E. coli* strain (Figure 2, Table 2). Probiotic treated pigs had vascular lesions in 56.7% (17/30) of ilea and 36.7% (11/30) of brainstems, whereas sham-treated pigs had vascular lesions in 63.3% (19/30) of ilea and 56.7% (17/30) of brainstems. There was a trend for probiotic treatment to reduce vascular lesions in brainstem (p = 0.098); however, the overall incidence of vascular lesions was not statistically significant between probiotic and sham treated groups for either tissue (NS, Fisher’s exact test).

## Discussion

The probiotic strain expressing globotetraose used in this study was designed specifically to bind and neutralize Stx2e, the toxic principal of piglet edema disease. This construct was effective at neutralizing 98.4% of Stx2e cytotoxicity in vitro [22]. The dose of probiotic and frequency of administration used here were extrapolated from previous studies with a similar probiotic expressing a surface Gb3 mimic in a mouse model of STEC infection [23]. Here we show that post-exposure treatment twice daily with the probiotic was effective at reducing the fecal Stx2e in STEC inoculated piglets. Fecal toxin titers were significantly reduced by day three of probiotic administration, and remained lower for several days. Reduction of intestinal Stx may be a first line of defense in HUS prevention for EHEC infected patients. Despite the favorable reduction in intestinal Stx, treatment with the receptor mimic probiotic failed to reduce the incidence of both vascular lesions in target organs and clinical disease in STEC inoculated swine, indicating systemic absorption of Shiga toxin still occurred. While not statistically significant, the incidence of vascular lesions in brainstems of probiotic treated pigs was less than that of sham treated pigs. This subtle trend towards lower disease incidence may warrant further studies to increase the power to experimentally detect lesion differences. Interestingly, those pigs that developed clinical disease within the probiotic group had, on average, higher fecal toxin titers than subclinical pigs within this group, with the average toxin titers of clinical pigs approaching those of the sham treated groups on days 2–6 post inoculation. This could indicate a failure of the probiotic to reduce toxin titers in those pigs that broke with clinical disease, despite an overall reduction in toxin titers within this treatment group.

In contrast to our findings here, several studies in mouse models have shown the efficacy of various probiotic strains at preventing disease caused by EHEC. Importantly, however, the probiotics used in these studies were administered prior to colonization. For example, Leatham et al. showed that certain commensal *E. coli* strains were efficacious at preventing growth of an *E. coli* O157:H7 strain in the streptomycin-treated mouse model of EHEC infection [28]. Similarly, certain bifidobacterial strains were able to prevent mortality in a gnotobiotic mouse model of EHEC infection when administered seven days prior to inoculation with the EHEC strain [29]. While these probiotics ameliorated disease caused by EHEC via inhibition of colonization, other probiotics are thought to confer protection via other mechanisms, such as suppression of virulence factor production or alteration of the host immune response [30,31]. For example, in a recent study by Eaton et al., treatment with the probiotic *Lactobacillus reuteri* suppressed disease in a mouse model of EHEC infection, but had only a minimal effect on colonization. The authors propose that the probiotic may have affected production of Stx2, but that further investigation was required to confirm the mechanism [31]. Based on these studies, we anticipated that the receptor mimic probiotic would be efficacious at preventing systemic disease in our pig model. However, one important difference between our study and those described above is the timing of probiotic administration. The probiotics used in the previous studies were most efficacious when administered prior to colonization with EHEC, and would therefore have limited therapeutic utility in the treatment of existing infections. The probiotic strain used in our study was given 1 day post STEC inoculation to investigate its efficacy in a post-exposure setting - similar to that which might be experienced during outbreaks of foodborne illness secondary to EHEC. Preemptive treatment of individuals during EHEC outbreaks may not be practical, given their sporadic nature. Our receptor mimic probiotic was designed specifically to bind Stx within the gut; therefore, we hypothesized that it would be useful as a post-exposure therapy and our experimental design reflected this. We speculate that the receptor mimic probiotic would likely have been more effective at preventing systemic disease had it been administered prior to STEC inoculation, similar to previous studies.

In mice, treatment with a similar probiotic expressing a Gb3 mimic was protective against fatal systemic complications of infection with human-derived EHEC [21]. There are several differences between the mouse studies and the current study that may have influenced treatment outcome. The affinity of the Gb4 mimic bacterium for Stx2e is not quite as high as that of the previously described Gb3 mimic for other members of the Stx family that was used in the mouse studies [22]. This reduced affinity may have resulted in sub-optimal retention of Stx2e in the gut lumen. A further disparity between these studies is the timing of probiotic treatment. In mice, treatment with the probiotic strain was initiated at the time of EHEC challenge. Our experimental design delayed treatment for 24 hours in an attempt to more closely mimic post-exposure therapy. As a result, pigs in this study were exposed to intestinal Stx2e prior to treatment with the probiotic. Pigs are known to be extremely susceptible to intravenous Shiga toxin (LD_50_ 3 ng/kg) [32]. It is likely that the early and continuous exposure to systemic toxin absorbed from the gut initiates an irreversible cascade of events leading to clinical disease development in some animals. This may represent an obstacle for post-exposure therapy in human beings because of the delay between the onset of diarrhea and diagnosis in EHEC infections [5]. Similarly, when probiotic treatment was delayed in the mouse model for 24 or 48 hrs, mice were not protected from death when challenged with the most virulent EHEC strain [23].

## Conclusions

In conclusion, the results of this study show that post-exposure treatment with a Stx2e-receptor mimic probiotic is effective at reducing Stx within the gut of a porcine model of STEC infection. Despite its efficacy at reducing gut Stx, the probiotic failed to prevent vascular necrosis in target organs and clinical disease, indicating systemic toxin absorption still occurred. Further investigation is warranted to determine if adjusting the time of treatment would have been more effective at ameliorating clinical STEC disease.

## Competing interests

The authors declare that they have no competing interests.

## Authors’ contributions

SJH contributed to animal studies, fecal toxin assessment, and evaluated histopathology; AH contributed to animal studies, fecal toxin assessment, and preparation of inoculation strains; JP and AP designed and created the probiotic and sham strains; NC contributed to study design and animal studies. SJH and NC were predominantly responsible for manuscript preparation and data analysis, although all authors contributed. All authors approved the final manuscript.
